# Independent Candidate Serum Protein Biomarkers of Response to Adalimumab and to Infliximab in Rheumatoid Arthritis: An Exploratory Study

**DOI:** 10.1371/journal.pone.0153140

**Published:** 2016-04-06

**Authors:** Ignacio Ortea, Bernd Roschitzki, Rosario López-Rodríguez, Eva G. Tomero, Juan G. Ovalles, Javier López-Longo, Inmaculada de la Torre, Isidoro González-Alvaro, Juan J. Gómez-Reino, Antonio González

**Affiliations:** 1 Laboratorio Investigacion 10 and Rheumatology Unit, Instituto de Investigacion Sanitaria - Hospital Clínico Universitario de Santiago, Santiago de Compostela, Spain; 2 Functional Genomics Center Zurich, University and ETH Zurich, Switzerland; 3 Rheumatology Unit, Hospital Universitario de La Princesa, Instituto Investigacion Sanitaria Princesa, Madrid, Spain; 4 Rheumatology Unit, Hospital General Universitario Gregorio Marañon, Madrid, Spain; 5 Department of Medicine, University of Santiago de Compostela, Santiago de Compostela, Spain; Nippon Medical School Graduate School of Medicine, JAPAN

## Abstract

Response to treatment of rheumatoid arthritis shows large inter-individual variability. This heterogeneity is observed with all the anti-rheumatic drugs, including the commonly used TNF inhibitors. It seems that drug-specific and target-specific factors lead individual patients to respond or not to a given drug, although this point has been challenged. The search of biomarkers distinguishing responders from non-responders has included shotgun proteomics of serum, as a previous study of response to infliximab, an anti-TNF antibody. Here, we have used the same study design and technology to search biomarkers of response to a different anti-TNF antibody, adalimumab, and we have compared the results obtained for the two anti-TNF drugs. Search of biomarkers of response to adalimumab included depletion of the most abundant serum proteins, 8-plex isobaric tag for relative and absolute quantitation (iTRAQ) labeling, two-dimensional liquid chromatography fractionation and relative quantification with a hybrid Orbitrap mass spectrometer. With this approach, 264 proteins were identified in all the samples with at least 2 peptides and 95% confidence. Nine proteins showed differences between non-responders and responders (*P* < 0.05), representing putative biomarkers of response to adalimumab. These results were compared with the previous study of infliximab. Surprisingly, the non-responder/responder differences in the two studies were not correlated (r_s_ = 0.07; *P* = 0.40). This overall independence with all the proteins showed two identifiable components. On one side, the putative biomarkers of response to either adalimumab or infliximab, which were not shared and showed an inverse correlation (r_s_ = -0.69; *P* = 0.0023). On the other, eight proteins showing significant non-responder/responder differences in the analysis combining data of response to the two drugs. These results identify new putative biomarkers of response to treatment of rheumatoid arthritis and indicate that they are notably drug-specific.

## Introduction

Rheumatoid arthritis (RA) is a chronic disease involving autoimmune reactivity and inflammation of multiple symmetric peripheral joints causing important disability and accompanied of other manifestations and significant life shortening [1]. Its evolution has been greatly improved by effective drugs that are globally known as disease-modifying antirheumatic drug (DMARD) [2]. They include recently developed target specific drugs, as the TNF inhibitors (TNFi) and other biologics jointly known as biological DMARD (bDMARD). Unfortunately, patients present large inter-individual variability in response to all the DMARD, independently of their target or molecular nature. This means that about a third of the patients starting treatment with a DMARD will not respond and will require change to a different one. This has motivated a lot interest in the finding of biomarkers for prediction of response [3]. Ideally, these biomarkers will discriminate between non-responders (NR) and responders (R) to a given DMARD. Unfortunately, we are very far from this panorama and some authors even question the possibility of such biomarkers, at least, in relation with the bDMARD [4]. According to these authors, biomarkers identify patients that fail to respond to any bDMARD, and therefore they will not be useful for guiding therapeutic choices. These ideas are disputable because differences between the drug molecules, their routes of administration and doses in addition to the molecular target could lead to specificity on biomarkers [5–8]. This drug-specificity is supported by the available evidence, which shows that most proposed biomarkers of prediction of response to treatment in RA are informative for some bDMARD but not for others. A notable example is RA seropositivity that has been informative for responses to the anti-CD20 monoclonal rituximab (RTX) and to the anti-IL6R antibody tocilizumab (TCZ), but not for response to abatacept, which inhibits T cell coestimulation, or to the TNFi [9–12]. Also, some of the genetic biomarkers seem to be informative for one of the TNFi, but not for the others [13–15]. With these antecedents, we considered interesting to compare putative biomarkers of response to two TNFi to see if they were redundant or independent. Therefore, we performed a shotgun proteomic discovery study of response to adalimumab (ADA) using exactly the same procedure we have applied previously for analyzing the response to infliximab (IFX) [16], and subsequently we compared the results obtained with these two anti-TNF monoclonal antibodies. This is necessary because there are not any shotgun proteomic study to identify predictive biomarkers in RA apart from two addressing response to IFX [16,17].

In this exploratory study, we have identified nine putative serum protein biomarkers of response to ADA and we have found that the patterns of protein differences between NR and R to ADA and to IFX are independent overall. The protein differences included drug-specific components and a common component. These results indicate that it will be possible to obtain biomarkers distinguishing response to these two bDMARD.

## Material and Methods

### Sample collection

Patients with RA that have not received before any bDMARD were invited to participate. Serum was collected in VACUETTE^®^ Z Serum Sep Clot Activator tubes (Greiner Bio-One), aliquoted and stored at −80°C before starting ADA administration. Response to treatment was assessed 6 months after ADA initiation according with the European League Against Rheumatism (EULAR) criteria [18]. These criteria are based in the Disease Activity Score 28 joints (DAS28), which is a composite index of disease activity including erythrocyte sedimentation rate, global patient health as self-reported, and counts of swollen joints and of tender joints in a given set of 28 joints. A score over 5.1 indicates high disease activity, whereas a score below 3.2 indicates low activity. R defined with the EULAR criteria show DAS28 ≤ 3.2 at 6 months and improvement from baseline > 1.2. On the contrary, NR show little improvement from baseline in DAS 28, ≤ 0.6, or if they show modest improvement, ≤ 1.2, they remain in high activity, DAS28 > 5.1, at 6 months. The intermediate group of moderate responders was not included in the current study to increase the chances of finding differences.

Four NR patients and four R were selected (Table 1) as eight is the maximum number of different isotopes available for iTRAQ labeling [19,20]. Sample collection was approved by the Hospital Universitario de La Princesa ethics committee. All patients gave their written informed consent.

**Table 1:** Main clinical and demographic characteristics of the patients with RA treated with ADA^a^.

Characteristic		1	2	3	4	5	6	7	8
Gender		w	w	m	w	m	w	w	w
Age (years)		30	65	36	49	70	43	34	41
TX gap (mo.)		25	42	42	124	137	126	56	62
RF		+	+	+	+	+	+	-	+
anti-CCP	status	-	+	+	+	+	-	+	+
	titer	< 25	1081	707	296	959	< 25	76	814
Erosions		+	-	-	+	+	-	+	+
Previous cDMARD		2	1	2	7	2	2	1	3
HAQ	base.	2.5	2.125	1.5	1.75	0.125	1.25	1	1.25
CRP	base.	9.73	1.47	2	1.89	2.44	0.35	1.51	0.74
DAS28	base.	6.7	5.7	5.1	5.5	4.1	6.2	5.1	5.6
	3 mo.	5.7	5.1	4.3	6	2.6	1.8	3.4	3.6
	6 mo.	5.8	5.2	6.5	5.2	2.6	1.1	2.1	2.2
EULAR	3 mo.	NR	NR	M	NR	R	R	M	M
	6 mo.	NR	NR	NR	NR	R	R	R	R

^a^ Abbreviations: w = woman; m = man; TX gap = time elapsed since disease starts and ADM treatment; mo = months; RF = rheumatoid factor; cDMARD = number of conventional disease modifying antirheumatic drugs administered before starting ADM treatment; HAQ = Health Assessment Questionnaire Disability Index; base. = baseline; CRP = C reactive protein; DAS28 = disease activity score 28 joints; EULAR = European League Against Rheumatism treatment response criteria; NR = non-responder; M = moderate responder; R = responder.

### Sample preparation

Serum samples were processed as described [16]. Briefly, the six most abundant proteins were depleted using the Hu-6 Multiple Affinity Removal System kit (Agilent Technologies, Wilmington, DE). Afterwards, proteins were reduced and cysteine was blocked with methyl mehanethiosulfonate followed by digestion with Sequencing Grade Modified trypsin (Promega, Madison, WI). Each digestion was labeled with one of the 8-plex iTRAQ reagents (AB Sciex, Framingham, MA) and all iTRAQ-labeled samples were combined into one tube. Peptides were separated in a PolyLC SCX Polysulphoethyl (PolyLC, Columbia, MD), on a high-pressure LC (HPLC) pump using a two-buffer system with gradient [16]. An aliquot of each fraction was desalted and analyzed by matrix-assisted laser desorption/ionization (MALDI)-time of flight (TOF)-TOF (4800, AB Sciex) mass spectrometry (MS) to check its peptide complexity. The poor fractions were discarded, and the other fractions were mixed according to their complexity.

### Nano-reverse-phase LC-MS/MS

The fractions prepared in the previous step were analyzed with nanoHPLC-MS/MS. The NanoLC-Ultra system (Eksigent, Dublin, CA) was used coupled to an Orbitrap Velos hybrid mass spectrometer (Thermo-Finnigan, San Jose, CA). The separation was performed on a tip column packed with Magic RP C18 AQ, 200A, 3 μm beads (Bischoff GmbH, Leonberg, Germany), with the previously described gradient [16]. The mass spectrometer was operated in data-dependent mode with the previously reported ion scanning parameters [16]. Fragmented peptide masses were set in dynamic exclusion for 60 s and singly charged ions were excluded from MS/MS analysis. To improve sensitivity of the MS/MS analysis for peptides of low-abundance proteins, each fraction was run a second time excluding previously fragmented precursors.

### Protein identification and protein relative abundance

Peptide and protein identification was performed with ProteinPilot software v4.0 (AB Sciex) and the Paragon algorithm [21]. Proteins having at least one peptide above the 95% confidence level were recorded. False discovery rates were estimated using a concatenated target-decoy database [22]. For the estimation of the protein abundance ratio, the intensities of iTRAQ reporter ions for each MS/MS spectra were extracted and the sum ratio for each protein was calculated across the spectra matched to the corresponding peptides. Data were normalized for loading error by bias corrections using ProteinPilot. The differences between R and NR were determined on the transformed data (arc sin hyperbolic) using the two‐tailed unequal variance t-test with Statistica 7.0 (StatSoft, Tulsa, OK) [23]. Threshold for significance was set at *P* < 0.05. No correction for the number of test was applied because it would lead to no differences by the very same design of the iTRAQ system that is limited to eight samples [19,20]. The ADA results were compared by non-parametric correlation analysis (Spearman rho) with the IFX results of our previous study [16], given the lack of normality of the distribution of *P* values. This comparative analysis was based on the *P* values of the NR/R contrast inside each experiment to retain the low technical variability of iTRAQ. Gene ontology analysis was performed in the AmiGO web application (http://amigo.geneontology.org/rte). For these analyses, the likely contaminants from sample processing as trypsin, keratins or latex proteins were excluded, as well as, uncharacterized proteins.

### Technical validation with ELISA

Independent assays for the nine proteins that showed differential abundance in the R/NR comparison in response to ADA were searched. For four of the proteins (TAGLN2, SH3BGRL3, TCFL5 and isoform 2 of TPM3) no commercial assay was found, for the other five proteins an ELISA was identified and used. They were the Human ABI family, member 3 (NESH) binding protein ELISA Kit (Mybiosource, San Diego, CA) for ABI3BP; the Human Tropomyosin alpha-4 chain (TPM4) ELISA kit (CUSABIO, Wuhan, Hubei Province, PRC); the ELISA Kit for Human Cofilin 1, Non Muscle (CFL1) (Cloud-Clone, Houston, TX); the Hemopexin (HPX) Human ELISA Kit (Abcam, Cambridge, UK); and the Complement C3 Human ELISA kit (Abcam). Assays were performed in duplicate according with the manufacturer instructions except for HPX, TPM4 and CFL1 that required extended incubation times to reach sufficient sensitivity. Concentrations in NR and R patients were compared after logarithmic transformation with Student t test.

## Results

The discovery proteomic analysis involved NR and R (moderate responders were excluded) patients after 6 months in treatment with ADA as the first bDMARD (Table 1). The serum samples were taken before starting ADA and processed as described in methods leading to the identification of 264 proteins with at least 2 peptides and 95% confidence (S1 Table). This large number included proteins of medium and low abundance in serum. Some examples in the ng/mL range were P-selectin, thioredoxin, mannan-binding lectin serine protease 2, insulin-like growth factor-binding protein 1 and soluble complement receptor 2. In the opposite extreme, concentrations reached the mg/mL level. Therefore, the experiment covered a dynamic range of up to 10^6^.

Gene ontology analysis of the identified proteins showed enrichment in proteins involved in response to stress, protein activation cascade and defense response as the three most notable biological processes. Other abundant processes were related with wound healing, complement activation and coagulation. Most of the proteins were of extracellular or vesicle location as expected for serum proteins. Regarding the molecular functions, they were particularly enriched in inhibitors of proteases and in transport functions either as protein or as metabolite binding proteins. These results were very similar to the found in our previous study of response to IFX [16].

**Fig 1:**
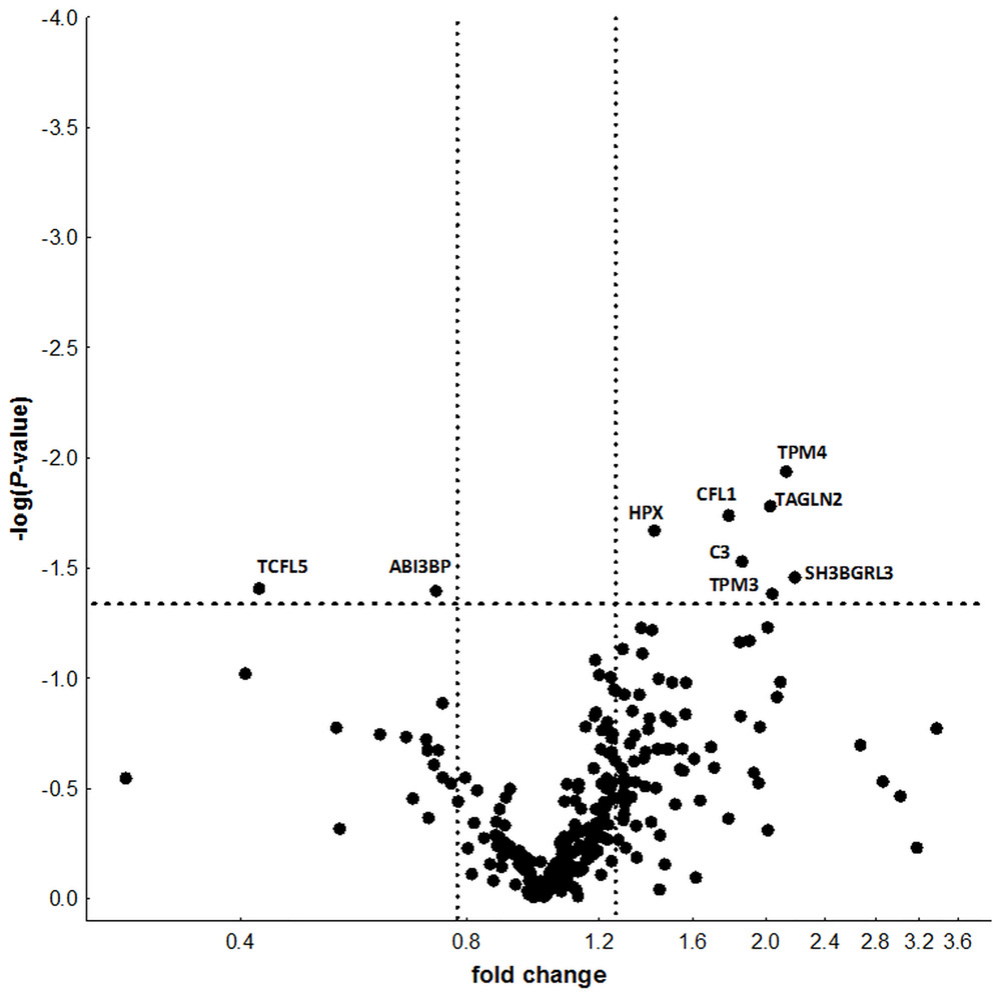
Volcano plot of protein relative abundances between NR and R patients in response to ADA. The X axis shows the NR/R fold change in logarithmic scale. The Y axis shows the–log of the *P* value. Horizontal dashed line corresponds to *P* = 0.05. Vertical dotted lines correspond to fold changes 0.77 and 1.3.

The iTRAQ label ratios were used for relative quantification of the 264 high confidence proteins and for comparison between NR and R patients (Fig 1 and S2 Table). In this way, we discovered nine proteins showing differential relative abundance in the two groups of patients. Seven of these proteins were more abundant in NR than in R patients (fold changes > 1.3), the other two were more abundant in R patients (fold changes < 0.77). To validate technically these results, commercial ELISA were done for the five proteins with available kits. Only one of the proteins, HPX, showed significant differences between NR and R patients (*P* = 0.037, two-tail t test). However, the five proteins showed the same direction of change observed with the iTRAQ analysis, which is unlikely at random (*P* = 0.031, one-tail binomial test).

**Fig 2:**
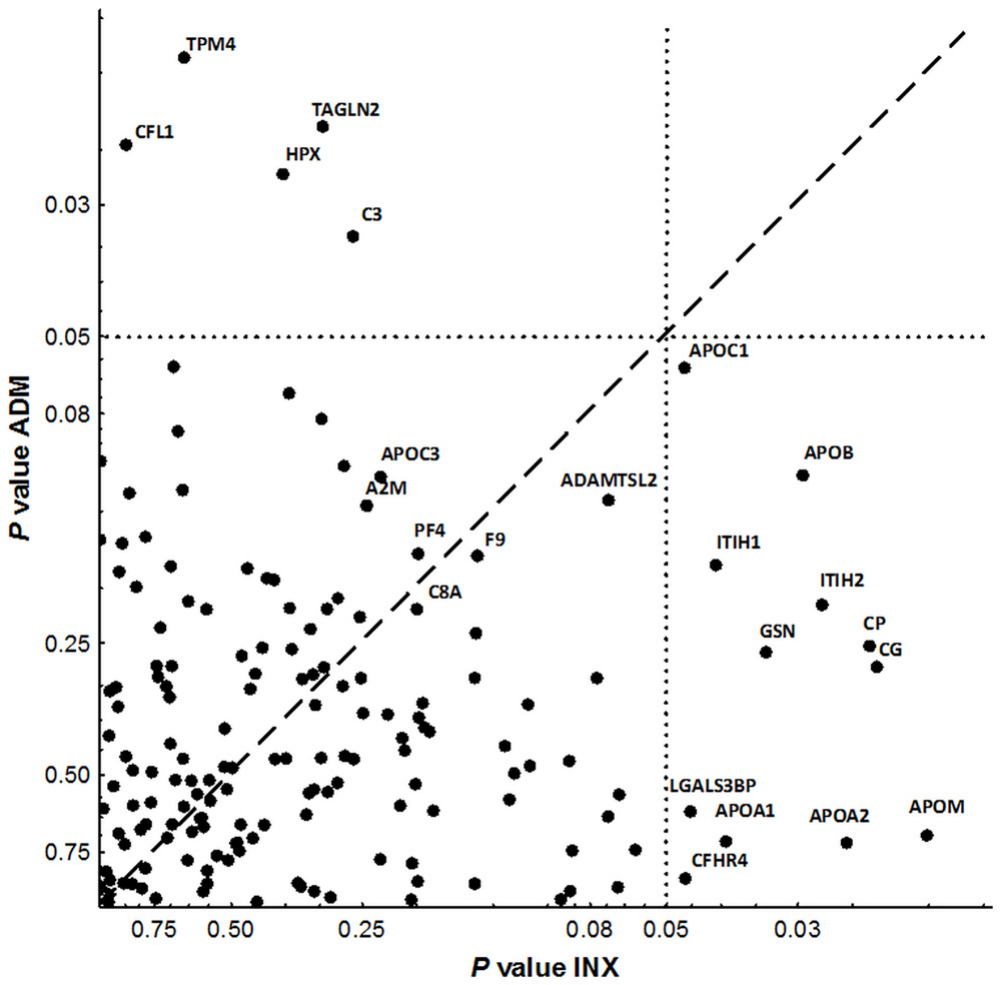
Scatter plot of *P* values comparing NR and R to IFX and to ADA. The 166 proteins quantified with confidence in all patients are shown. Only symbols of significantly different proteins between NR and R patients in any of the analyses (IFX- and ADA-specific, and in the combined IFX+ADA analysis) are shown.

These results were compared with our previous study of response to IFX, which involved an independent set of eight patients [16]. When these results were analyzed in exactly the same way as the ADA results, they showed 17 putative biomarkers of response (see below). For the comparison between responses to the two TNFi, only the 166 proteins quantified with high confidence in all the subjects from the two studies were included. Unfortunately, some of the putative biomarkers were among the proteins not identified across all samples, and therefore, nine proteins differentiating NR/R in the ADA or the IFX analyses were not available (four in response to ADA missing in the IFX experiment, and five in response to IFX missing in the ADA experiment). The comparison confronted the *P* values for the NR/R analysis in patients treated with ADA with the *P* values for the patients treated with IFX (Fig 2). Surprisingly, the results were independent as shown by the lack of correlation of the *P* values (r_s_ = 0.07; *P* = 0.40). This was also shown by the lack of any protein in the upper quadrant of Fig 2, which should have included any proteins significantly different between NR and R patients to ADA and to IFX. Even more notable, the 17 proteins that were significantly different in NR/R comparisons either in the ADA (5) or the IFX (12) analysis showed a significant inverse correlation (r_s_ = -0.69; *P* = 0.0023). However, it should be noted that there were some proteins showing similar *P* values for the NR/R contrast with the two TNFi (near the middle line in Fig 2). Therefore, it seemed possible that the overall independence of the results included several components.

Trying to highlight the concordant component observed in Fig 2, we combined the results of the two studies. This analysis was done by comparing the protein relative abundances of the eight NR patients (the four NR to ADA plus the four NR to IFX) with those of the eight R patients (the four R to ADA plus the four R to IFX). Eight proteins, ADMATSL2, A2M, APOB, APOC3, F9, C3, C8A and PF4, were significantly different in this analysis (Table 1). Two of these proteins, C3 and APOB, were significantly different in the drug-specific analyses with ADA and IFX, respectively. The other six proteins were new as putative biomarkers. They were among the proteins showing similar *P* values in the drug-specific analyses (identified by their symbols in the lower left quadrant of Fig 2).

We have continued analysis to summarize all results and select proteins for future validation, which is very necessary given the exploratory nature of our results. Initially the summary list included 32 putative biomarkers, but three proteins were excluded because they showed high inter-individual variability both within NR and within R patients. They were vitamin D-binding protein (GC), ceruloplasmin (CP) and TAGLN2. In the case of GC, this variability was very likely due to a well-known common polymorphism [24], because two alleles of this protein showed opposite NR/R changes (accessions D6RAK8 and P02774). The remaining 29 putative biomarkers are divided in two groups: drug-specific biomarkers and shared biomarkers. The drug-specific biomarkers are the remaining eight proteins identified in the ADA analysis (after excluding TAGLN2) and the 15 proteins differentiating NR and R patients in response to IFX (after excluding GC and CP). These later proteins include 12 already reported putative biomarkers [16], and APOA1, APOC1 and LGALS3BP. These three proteins were significantly different in the NR/R analysis with the two‐tailed unequal variance t-test used here, but no with the equal variance t-test used previously. The group of shared biomarkers includes the eight proteins described in the previous paragraph, which were significantly different in the comparison across IFX and ADA. This list of 29 proteins for replication included two that we have already replicated here. These two biomarkers, APOA1 and PF4, were the only proteins identified as associated with response to IFX in the unique shotgun proteomic study done by other authors [17], although the direction of change of APOA1was the contrary to the previously reported. Finally, it is worth to note that no protein showed significant opposite changes with the two TNFi.

## Discussion

The most notable result of our exploratory study has been the independent patterns of protein abundance differences between NR and R patients in response to either IFX or ADA. Other interesting results have been the confirmation of the feasibility of the approach for the relative quantification of many proteins in serum, and the preliminary identification of putative biomarkers for predicting response to ADA and others that will inform also of response to IFX. All these candidate biomarkers require replication given the exploratory nature of shotgun proteomic studies and the small number of samples included here.

There has been interest in knowing if the various TNFi show differences in efficacy in spite of all them inhibiting the same target. The arguments favoring the existence of differences are based in the structure of the molecules, their immunogenicity, pharmacodynamics, interactions with other molecules modulating their action as the FcγRs and in differences in their affinity for the TNF target [7,8]. The differences in clinical efficacy are manifest between etanercept (ETC), which is a soluble TNF receptor, and the monoclonal antibodies, IFX and ADA in the treatment of other autoimmune diseases. ETC shows poor efficacy for inflammatory bowel disease, psoriasis and the uveitis observed in spondyloarthritis patients, whereas IFX or ADA show good results in these three situations [7,8,25,26]. However, there are not yet convincing evidence of differences, at the group level, in their clinical efficacy to treat RA. This lack of differences in RA could be due to the lack of appropriate studies or to inter-individual variability. For example, the good correlation between the presence of anti-drug antibodies and poor response to TNFi has been demonstrated at the individual patient level and ETC is markedly less immunogenic than ADA and IFX [27–30], however we have only a few analyses showing better response at the group level with ETC than with the other two TNFi [31,32]. In any case, the widespread experience of patients with RA that respond to a TNFi after failing to a previous TNFi is a powerful argument supporting the differences between these drugs at the individual patient level [33,34].

More generally, when all bDMARDs are considered, some authors think that the broadly similar efficacy at the group level, as the studied in clinical trials, excludes the possibility of drug-specific or target-specific biomarkers [4]. However, the similar efficacy of the bDMARDs does not apply to the individual patients, as already commented, and could be more apparent than real at the group level. In effect, there is only a clinical trial comparing head-to-head different bDMARDs and it showed superiority of TCZ monotherapy over ADA monotherapy [35]. Also, the indirect comparisons between clinical trials, although less convincing than direct head-to-head studies, have shown differences even between the various TNFi [31,32]. In any case, biomarkers are not directly affected by overall efficacy at the group level because they should be useful at the individual level, where it is common to observe a good response after switching to a different bDMARD [33,34,36–42].

In addition, some biomarkers of differential response to bDMARD have already emerged. For example, two different synovial phenotypes were associated with response to either ADA or to TCZ [43]. In other studies, an increased interferon signature in blood cells was associated with good response to TCZ [44], and with poor response to RTX [45,46]; and several genetic biomarkers are specific of particular TNFi [13–15]. Finally, RA seropositivity affects response to RTX and to TCZ, but not to TNFi or abatacept [9–12], as already mentioned.

The putative protein biomarkers of response to IFX and to ADA were not completely divergent. There were some proteins showing a degree of concordance, including the eight proteins highlighted in the combined analysis. In addition, there were no proteins with opposed direction of change in response to the two TNFi. These results are based in a large panel of proteins and, consequently, not sensitive to artefacts of specific proteins. Even so, replication is necessary for confirmation.

Replication will be also the next step in the validation of the putative biomarkers. A focused proteomics technology, as selected reaction monitoring (SRM), will be an efficient approach for replication because it allows the quick analysis of all the candidate biomarkers in many samples. With SRM validation in mind, we generate a list of 29 proteins of interest. This list could be covered in a single experiment aiming to obtain accurate results with three transitions per peptide and two peptides per protein to a total of 174 transitions.

Potential biomarkers for response to IFX were enriched in apolipoproteins, members of the complement pathway and acute phase reactants [16]. In contrast, potential biomarkers of response to ADA include only C3 in the complement pathway and not any apolipoprotein or acute phase reactant. There was a new pathway including three proteins, TPM4, TPM3 and CFL1 that are related with actin cytoskeletal organization. Another protein of interesting function is SH3BGRL3, which causes resistance to apoptosis induced by TNF in normal and cancer cells [47]. However, all these potential biomarkers require confirmation.

One of the strengths of this study is the approach followed for biomarker identification. The iTRAQ technology reduces technical variability by including the eight samples in the same assay [19,20]. Depletion of the highest abundance serum proteins and intensive fractionation allow for sensitive identification of proteins along a wide range of concentrations and properties. In this respect, it is notable that several low molecular weight proteins (9–11 kDa) as APOC1, SH3BGRL3, APOA2 and APOC3, were quantified given that they are commonly lost in other proteomic approaches. Finally, the use of MS/MS and the strict criteria we have applied, requiring two peptides per protein with a confidence over 95%, sensitive statistical analysis and exclusion of proteins with excessive inter-individual variability have likely contributed to a list of candidate biomarkers with good chances of validation in the necessary replication studies. In this regard, our study has already replicated the only agnostic proteomic study of response to IFX in RA done by other authors [17], as already mentioned. Limitations of the study include the lack of validation in independent samples and the possibility of confounding by factors that could be unrelated with the treatment. However, we have already provided some technical replication of the results with ELISA of five of the putative biomarkers of response to ADA, as shown here, and strong replication of the putative biomarkers of response to IFX and of the putative biomarkers to IFX + ADA mentioned in Table 2. In effect, we have been able to study 11 of the 16 proteins associated with response to IFX, and 6 of the 8 proteins associated with response to IFX + ADA in sera of 26 independent RA patients (14 R and 12 NR) by SRM proteomics (Ortea, N. et al. *Manuscript in preparation*). This analysis showed replication of 7/11 proteins and 4/6 proteins in each of the two categories (*P* < 0.05). Regarding the possibility of confounding, none of the demographic or clinical data available showed notable differences between R and NR patients. The most questionable was baseline HAQ, which was significantly lower in R than in NR patients (*P* = 0.02) but was not accompanied by other factors associated with severity or chronicity of RA as DAS28, erosions, seropositivity or time since disease onset (Table 1).

**Table 2:** Summary of putative biomarkers from the ADA and IFX analyses^a^.

Accession	Name	Symbol	IFX^b^	ADA	IFX+ADA
Q86TH1	ADAMTS-like protein 2	ADAMTSL2			+
Q9HDC9	Adipocyte plasma membrane-associated	APMAP	+		
P01023	Alpha-2-macroglobulin	A2M			+
P02647	Apolipoprotein A-I	APOA1	+		
P02652	Apolipoprotein A-II	APOA2	+		
P04114	Apolipoprotein B-100	APOB	+		+
P02654	Apolipoprotein C-I	APOC1	+		
P02656	Apolipoprotein C-III	APOC3			+
O95445	Apolipoprotein M	APOM	+		
P00740	Coagulation factor IX	F9			+
E9PK25	Cofilin-1	CFL1		+	
P01024	Complement C3	C3		+	+
Q6U2E9	Complement C4-B alpha chain	C4B	+		
P07357	Complement component C8 alpha chain	C8A			+
C9J7J7	Complement factor H-related protein 4	CFHR4	+		
Q08380	Galectin-3-binding protein	LGALS3BP	+		
P06396	Gelsolin	GSN	+		
P02790	Hemopexin	HPX		+	
P19827	Inter-alpha-trypsin inhibitor heavy chain H1	ITIH1	+		
P19823	Inter-alpha-trypsin inhibitor heavy chain H2	ITIH2	+		
P06753-2	Isoform 2 of Tropomyosin alpha-3 chain	TPM3		+	
P02751-7	Isoform 7 of Fibronectin	FN1	+		
O00187	Mannan-binding lectin serine protease 2	MASP2	+		
P02776	Platelet factor 4	PF4			+
Q9H299	SH3 domain-binding Glu-rich-like 3	SH3BGRL3		+	
D3YTG3	Target of Nesh-SH3	ABI3BP		+	
P07996	Thrombospondin-1	THBS1	+		
F8W9A4	Transcription factor-like 5 protein	TCFL5		+	
P67936	Tropomyosin alpha-4 chain	TPM4		+	

^a^ Three additional proteins were different in at least one of the analyses, D6RAK8, P00450 and P37802, but they were excluded because they showed large inter-individual variability

^b^ IFX = showing significant differences between NR and R patients treated with infliximab; ADA = idem treated with adalimumab; IFX+ADA = idem treated with either IFX or ADA

## Conclusions

New putative biomarkers of response to ADA were identified in a discovery phase of study. The putative biomarker proteins and the whole panel of differences between NR and R patients after treatment with ADA were independent of the panel observed after treatment with IFX. These results suggest that protein serum biomarkers will be able to inform the election between these two TNFi and, probably, also in relation with other bDMARDs. A list of the candidate biomarkers was produced for replication in future studies with focused proteomic technologies.

## Supporting Information

S1 Table Proteins identified in the serum of patients with RA treated with ADA. (XLS)

S2 Table Proteins significantly different between NR and R patients with RA treated with ADA. (XLS)

S3 Table Raw untransformed protein relative abundances for each of the patients treated with ADA. (XLS)
